# Management factors affecting adrenal glucocorticoid activity of tourist camp elephants in Thailand and implications for elephant welfare

**DOI:** 10.1371/journal.pone.0221537

**Published:** 2019-10-01

**Authors:** Pakkanut Bansiddhi, Janine L. Brown, Jaruwan Khonmee, Treepradab Norkaew, Korakot Nganvongpanit, Veerasak Punyapornwithaya, Taweepoke Angkawanish, Chaleamchat Somgird, Chatchote Thitaram

**Affiliations:** 1 Center of Elephant and Wildlife Research, Chiang Mai University, Chiang Mai, Thailand; 2 Department of Companion Animals and Wildlife Clinics, Faculty of Veterinary Medicine, Chiang Mai University, Chiang Mai, Thailand; 3 Center for Species Survival, Smithsonian Conservation Biology Institute, Front Royal, VA, United States of America; 4 Department of Veterinary Bioscience and Veterinary Public Health, Faculty of Veterinary Medicine, Chiang Mai University, Chiang Mai, Thailand; 5 Department of Food Animal Clinic, Faculty of Veterinary Medicine, Chiang Mai University, Chiang Mai, Thailand; 6 Excellent Center of Veterinary Public Health, Chiang Mai University, Chiang Mai, Thailand; 7 National Elephant Institute, Lampang, Thailand; University of Tasmania, AUSTRALIA

## Abstract

Elephant camps are among the most popular destinations in Thailand for tourists from many countries. A wide range of management strategies are used by these camps, which can have varied impacts on health and welfare of elephants. The objectives of this study were to examine relationships between FGM (fecal glucocorticoid metabolite) concentrations and camp management factors (work routine, walking, restraint, rest area, foraging), and to other welfare indicators (stereotypic behaviors, body condition, foot health, and skin wounds). Data were obtained on 84 elephants (18 males and 66 females) from 15 elephant camps over a 1-year period. Elephants were examined every 3 months and assigned a body condition score, foot score, and wound score. Fecal samples were collected twice monthly for FGM analysis. Contrary to some beliefs, elephants in the observation only program where mahouts did not carry an ankus for protection had higher FGM concentrations compared to those at camps that offered riding with a saddle and shows. Elephants that were tethered in the forest at night had lower FGM concentrations compared to elephants that were kept in open areas inside the camps. There was an inverse relationship between FGM concentrations and occurrence of stereotypy, which was not anticipated. Thus, assessing adrenal activity via monitoring of FGM concentrations can provide important information on factors affecting the well-being of elephants. Results suggest that more naturalistic housing conditions and providing opportunities to exercise may be good for elephants under human care in Thailand, and that a no riding, no hook policy does not necessarily guarantee good welfare.

## Introduction

Elephant camps are among the most attractive destinations in Thailand for tourists from many countries. A wide range of management strategies are used by these camps, which can have varied impacts on health and welfare of elephants [1, 2]. Thus, it is increasingly important to objectively answer questions about how specific tourist activities and camp management practices affect physical and physiological function of individual elephants.

Glucocorticoids (GCs) are released in response to a range of stimuli through activation of the hypothalamic-pituitary adrenal (HPA) axis, and are routinely measured in the assessment of animal welfare and stress [3, 4]. In elephants, GCs increase in response to normal physiological changes; i.e., pregnancy, parturition, and musth [5–8] and also to more adverse stressful conditions; i.e., human interactions and episodic loud noises [9], opening of a zoo to the public for the first time [10], high exhibit attendance [11], introduction of an unfamiliar conspecific [12], participating in public festivals and processions [8], transportation and relocation [9, 13–15], during the logging season [16, 17], housing in small enclosures [18], and during construction [19]. While many of these studies evaluated changes in circulating cortisol concentrations, in recent years, more researchers are employing fecal GC metabolite (FGM) analyses because sample collection is noninvasive. Feces can be collected easily and frequently, and sampling is feedback free because there is no need to capture and handle the animal. In addition, fecal samples are less affected by episodic fluctuations or pulsatility of hormone secretion [20, 21].

We acknowledge, however, that while elevations in GCs may be a sign of poor welfare, unchanged or even reduced activity can also be associated with chronic stress, making proper interpretation of GC data difficult [22]. For this reason, it is important to assess GCs in conjunction with other welfare measures, like those associated with behavior and health. Stereotypies are the most described welfare-related behaviors in captive elephants, which are repetitive, invariant behavior patterns with no obvious goal or function [23–25]. Health assessments have involved measures of body condition [26–28], metabolic function [29–32], and foot health [33, 34], and identified the importance of good diets, adequate exercise and soft substrates. One health problem associated with working conditions in Thailand is wounds, particularly those associated with riding and restraint equipment, and improper use of tools such as the ankus (i.e., hook).

Recently, we evaluated relationships between management factors and body condition, foot health, and skin wounds in this same population of captive elephants in Thailand [35]. The majority had body condition scores indicative of being overweight or obese, and mild foot problems, particularly rear foot cracks. Higher wound scores were observed in about a quarter of the cases where mahouts carried an ankus, and in association with being kept on hard, compact dirt substrates at night. Findings emphasized how camp management and associated activities can affect welfare outcomes; however, to date, there have been no large scale studies to examine the effect of these management practices on FGM concentrations in tourist elephants. Thus, the objectives of this study were to identify: 1) relationships between camp management factors and FGM concentrations; and 2) relationships between FGM concentrations and other welfare indicators: stereotypic behaviors, body condition, foot health, and skin wounds.

## Materials and methods

### Study animals

This study was approved by the Institutional Animal Care and Use Committee, Faculty of Veterinary Medicine, Chiang Mai University, Chiang Mai, Thailand (FVM-ACUC; S43/2559). Data were obtained on 84 healthy elephants (18 males and 66 females) from 15 elephant camps in the Chiang Mai area (Table 1), which were a subset of our previous study [35]. Elephant age ranged from 5 to 65 years and averaged 27.94±3.11 years for males and 35.53±1.34 years for females. We excluded data from musth bulls and known pregnant elephants because of physiological changes in cortisol that could bias results [36]. Musth elephants were identified by temporal gland swelling and secretion, and urine dribbling, while pregnant females were those that gave birth during the study period. Elephants were involved in five types of work or tourist activities: riding with a saddle; riding bareback; no riding but some tourist interactions; observation only; and elephant shows, as described by Bansiddhi et al. [2] and summarized in Table 1.

**Table 1:** Summary of elephants and mean (± SEM) fecal glucocorticoid metabolite (FGM) concentrations of elephants at 15 elephant camps in Chiang Mai province.

Camp No.	Total Number in Camp	N of Participating Elephants			N of Elephants for Each Type of Work					FGM (ng/g)
		Male	Female	Total	Riding with a Saddle	Riding Bareback	No Riding	Observation	Show	
1	46	3	7	10		10				69.04±2.13^a^
2	6	0	2	2	2					64.27±5.43^ab^
3	5	1	3	4		4				60.88±2.63^ab^
4	65	0	7	7			3	4		57.90±2.09^bc^
5	9	0	5	5		4	1			57.86±2.77^bc^
6	6	2	2	4	1	2			1	57.82±3.63^bcd^
7	10	1	5	6		3	3			57.32±2.61^bcd^
8	35	3	7	10		10				56.22±2.43^bcd^
9	4	0	3	3			3			51.09±3.58^bcde^
10	66	3	4	7	7					50.86±2.18^bcde^
11	5	0	4	4			4			48.87±3.35^bcde^
12	15	0	4	4		4				44.89±2.42^cde^
13	76	1	3	4	4					44.76±2.44^de^
14	7	1	4	5			5			41.8±1.88^e^
15	52	3	6	9	8				1	39.22±1.21^e^
Total		18	66	84	22	37	19	4	2	

^a-e^Different superscripts for FGM concentrations indicate significant differences (*P* < 0.05).

### Management survey

Data on elephant management were surveyed from direct observations and the management and medical records. Types of work, rest areas, foraging, and using an ankus were documented based on direct observations. Sex, age, duration of work, walking distance and time, and duration of chaining were surveyed from the management and medical record. Observers were veterinarians experienced in working with captive elephants from the Faculty of Veterinary Medicine, Chiang Mai University (CMU).

### Health and behavioral parameters

Elephants were examined every 3 months for 1 year for a total of four evaluations, and each was given a body condition score (BCS), foot score (FS), and wound score (WS). Briefly, we used the 5-point BCS scale developed by Morfeld et al. [27], with 1 representing the lowest and 5 representing the highest levels of body fat. Foot health was scored using a scale adapted from Harris et al. [37] and the British and Irish Association of Zoos and Aquariums (BIAZA)’s Elephant Welfare Group described by Todd [38]. Each foot was given a score of 0 (no problem), 1 (mild problems), 2 (moderate problems) or 3 (severe problems). The overall score of each elephant was the highest score from all four feet as described by Todd [38] (S1 Table). The wound score scale was developed by Schein et al. [39]. Each elephant was given a score of 0 (no wounds), 1 (minor wounds), or 2 (major wounds) (S2 Table). Minor wounds were those caused by the sharp hook of an ankus, or mild abrasions where there was mild bleeding but no infection; major wounds were penetrating injuries caused by a knife, ankus or other sharp object, and included lacerations, ulcers, and abscesses. At the time of each examination, current management and work activity information, and the presence of stereotypic behaviors (Yes or No) were recorded for each elephant.

### Fecal sample collection and FGM analysis

Fecal samples (~50 g) were collected twice monthly for 1 year through mixing of 2–3 central portions of fresh fecal boluses. The samples were placed in plastic bags and frozen within 1 hour of collection at -20°C until extraction and hormone analysis.

For extraction, all chemicals were obtained from the Sigma Chemical Company (St. Louis, MO), unless otherwise stated. The fecal extraction method was based on Brown et al. [40] and Norkaew et al. [31]. Briefly, wet fecal samples were dried in a conventional oven at 60°C for ~24–48 h and stored at -20°C until extraction. Frozen dried fecal samples were thawed at room temperature (RT), mixed well and 0.1 g (±0.01) of dry powdered feces placed in a glass tube containing 90% ethanol in distilled water. Samples were extracted twice by boiling in a water bath (96°C) for 20 minutes and adding 100% ethanol as needed to keep from boiling dry. Samples were centrifuged at 1,500 x g for 20 min, and the combined supernatants dried under air in a 50°C water bath. Dried extracts were reconstituted by vortexing for 1 min in 3 ml ethanol, dried again, and then diluted and vortexed in methanol for analysis. Extracts were stored at –20°C until EIA analysis.

Concentrations of FGM were determined using a double-antibody enzyme immunoassay (EIA) with a polyclonal rabbit anti-corticosterone antibody (CJM006) that has been validated for Asian elephants [31, 41]. Second antibody-coated plates were prepared by adding 150 μl of anti-rabbit IgG (0.01 mg/ml) to each well of a 96-well microtiter plate, and incubating at RT for 15–24 h. The wells were then emptied and blotted dry, followed by adding 250 μl blocking solution and incubating for 15–24 h at RT. After incubation, all wells were emptied, blotted and dried at RT (Sanpla Dry Keeper, Sanplatec Corp., Auto A-3, Japan) with loose desiccant in the bottom. After drying (humidity <20%), plates were heat-sealed in a foil bag with a 1g desiccant packet, and stored at 4°C until use.

Samples (50 μl), diluted 1:3 in assay buffer, or corticosterone standards (50 μl) were added to appropriate wells of pre-coated plates. Corticosterone-horseradish peroxidase (25 μl) was immediately added to each well, followed by 25 μl anti-corticosterone antibody added to all but nonspecific binding wells, and incubated at RT for 1 h. Plates were then washed four times with buffer (1:20 dilution, 20X Wash Buffer Part No. X007; Arbor Assays, MI) and 100 μl of TMB substrate solution added, followed by incubation for 45–60 min at RT without shaking. The absorbance was measured at 405 nm by a microplate reader (TECAN, Sunrise microtiter plate reader, Salzburg, Austria). Assay sensitivity (based on 90% binding) was 0.14 ng/ml. Samples were analyzed in duplicate; intra- and inter-assay CVs were <10% and <15%, respectively.

### Statistical analysis

Median and percentage data were calculated for stereotypic behaviors, BCS, FS, and WS. Descriptive FGM concentration data and elephant age are presented as the mean ± standard error of the mean (SEM). To compare means for more than two groups, one-way analysis of variance was used and a non-parametric Kruskal–Wallis rank sum test was used when the data did not meet either the assumption of normality or the assumption of equal variances.

Generalized estimating equations (GEE) were conducted for fitting marginal regression models using an R program [42], package geepack, function geeglm, under the optimal AR(1) correlation structure [43]. FGM concentrations were averaged quarterly around the time of each elephant and camp evaluation to assess relationships to management factors (Table 2) and the other welfare indicators: stereotypic behaviors, BCS, FS, and WS. Camps were treated as random effects. Univariate analysis was conducted and any variable having a significant univariate test at *P* < 0.15 was selected as a candidate for the multivariable analysis, statistical significance for which was set at *P* < 0.05. Pearson correlation tests were used to determine associations between continuous variables in the univariate model. Sex and Age were included in multivariable model as confounders.

**Table 2:** Description of demographic and management variables used in the GEE analysis.

Variable Name	Description	
Sex	Female or male	
Age	Age of elephant (years)	
Work Type	Type of work: observation, riding with a saddle, riding bareback, no riding, or show	
Work Hour	Duration of work when elephants interacted with tourists per day (h)	
Walk Distance Day	Walking distance during working period per day (m)	
Walk Time Day	Walking time during working period per day (min)	
Chain Hour	Duration of chaining per day (h)	
Rest Day	Rest areas during the day:	
	Shed:	Roofed structure used to keep several elephants in the same place, often restrained by ropes or chains
	Open area:	Elephants were free to walk, swim or forage on walking trails, grass fields, pastures, large dirt areas, or rivers or ponds
	Tree:	Elephants tied under trees inside the camps for shade
Rest Night	Rest areas during the night:	
	Shed:	Same as during the day
	Open area:	Unsheltered areas where elephants could be restrained apart from each other
	Enclosure:	Roofed structures built of metal or wood to house one or two elephants. Elephants were allowed free movement or tied to an internal post
	Forest:	Local forest land where elephants could be restrained
	Tree:	Same as during the day
Free foraging	Ability to forage in the forest or grass field everyday: Yes or No	
Ankus	Using an ankus to control an elephant: Yes or No	

For the GEE analysis of management factors, the final model was selected based on the smallest quasi-likelihood under the independence model criterion (QIC) values [44] using package MuMIn, function dredge [45]. Reference categories were chosen based on specific questions. For Sex, we chose male to determine how females, which generally are tamer, differ from males. For Work Type, we chose Observation because animal activists claim that those activities are the only ethical choice. For Rest Day and Rest Night, we had no specific comparison, so we used the largest category as the reference. For Free Foraging, we chose Yes because it is gaining in popularity and assumed to provide better welfare. For Ankus, we chose Yes because we wanted to see how ‘no ankus’, a new trend, differed from the more traditional ‘use of ankus’.

For GEE model of relationships between FGM and other welfare indicators, all variables in the multivariable analysis were included in the final model. Tukey's multiple comparisons of least-squares mean (LS-mean) were used as post hoc tests to determine differences between variable categories. In another analysis, BCS, FS, and WS were set as ordered factors and reported by orthogonal polynomial contrasts to examine increasing or decreasing severity within each factor.

## Results

### Descriptive statistics of health and behavioral parameters

Data (n = 324 observations) were obtained from 84 elephants, although some elephants had missing data points during some evaluation periods. Median BCS was 4 (range 2–5). The majority of elephants were BCS = 4, and none were BCS = 1. Median FS was 1 (range 0–3), which also was the mean. Median WS was 0 (range 0–2), with the majority of elephants having no visible wounds (Table 3). Wounds were noted in 42 elephants across 65 observations, caused by an ankus (63% of the observations, n = 41), knife (6%, n = 4), scratches (11%, n = 7), pressure sore (9%, n = 6), multiple causes (8%, n = 5), and unknown (3%, n = 2). Twenty-five percent (n = 21) of elephants presented stereotypic behaviors and 75% (n = 63) did not.

**Table 3:** Percentage of elephant body condition, foot health, and wound score categories from quarterly scoring periods over a 1-year period.

Score	Category	Time 1		Time 2		Time 3		Time 4		Total	
		n = 77	%	n = 81	%	n = 84	%	n = 82	%	n = 324	%
Body Condition Score	1	0	0.0	0	0.0	0	0.0	0	0.0	0	0.0
	2	1	1.3	2	2.5	5	6.0	5	6.1	13	4.0
	3	20	26.0	21	25.9	20	23.8	21	25.6	82	25.3
	4	41	53.2	41	50.6	39	46.4	31	37.8	152	46.9
	5	15	19.5	17	21.0	20	23.8	25	30.5	77	23.8
Foot Score	0	23	29.9	32	39.5	32	38.1	35	42.7	122	37.7
	1	30	39.0	30	37.0	35	41.7	40	48.8	135	41.7
	2	23	29.9	16	19.8	16	19.0	6	7.3	61	18.8
	3	1	1.3	3	3.7	1	1.2	1	1.2	6	1.9
Wound Score	0	55	71.4	60	74.1	73	86.9	71	86.6	259	79.9
	1	16	20.8	16	19.8	8	9.5	8	9.8	48	14.8
	2	6	7.8	5	6.2	3	3.6	3	3.7	17	5.2

### Factors associated with FGM concentration

A total of 1,751 fecal samples were collected and analyzed. Overall mean FGM concentration was 53.49±0.68 ng/g (range, 8.15–291.55 ng/g), and varied significantly across camps (Kruskal-Wallis chi-square = 223.17, df = 14, *P* < 0.01) (Table 1). There were differences in FGM concentration due to wounds (*F*_5,59_ = 2.39, *P* = 0.049). Elephants that had wounds caused by multiple causes had the highest FGM concentrations (74.12±5.26 ng/g) followed by elephants that had wounds caused by knife (56.57±6.26 ng/g), ankus (55.09±2.93 ng/g), unknown (53.10±3.63 ng/g), pressure sore (50.71±2.88 ng/g), and scratches (41.53±3.48 ng/g).

Results of univariate and multivariable GEE analyses of demographic and management variables associated with FGM are presented in Table 4. Variables significantly affecting FGM in the univariate analysis were: *Sex*, *Age*, *Work Type*, *Work Hour*, *Walk Distance Day*, *Walk Time Day*, *Rest Day*, and *Rest Night*. None of the correlations between continuous variables in the univariate model were strong; i.e., > 0.7 or > -0.7 [46] and so all were used in the multivariable model (S3 Table). From the multivariable analysis, factors that had the greatest effect on FGM in the final model included *Sex*, *Age*, *Work Type*, *Work Hour*, *Rest Day*, and *Rest Night*. Longer durations of work were associated with lower FGM. In the final model, *Age* and *Rest Day* were not significant, although a negative relationship between *Age* and FGM approached significance (*P* = 0.059).

**Table 4:** Univariate and multivariable GEE analyses of demographic and management variables associated with fecal glucocorticoid metabolite (FGM) concentrations.

Variable		N	Univariate analysis			Multivariable analysis		
			Estimate	SE	*P* value	Estimate	SE	*P* value
**Sex**								
	Male	18	Reference					
	Female	66	-8.400	2.640	0.002	-8.815	2.686	0.001
**Age**			-0.227	0.078	0.003	-0.175	0.093	0.059
**Work Type**								
	Observation	4	Reference					
	Riding with a saddle	22	-7.289	3.230	0.024	-16.900	6.490	0.009
	Riding bareback	37	0.407	3.077	0.895	-14.016	6.236	0.025
	No riding	19	-2.935	3.284	0.371	-7.544	3.566	0.034
	Show	2	-4.959	5.367	0.356	-34.232	7.616	<0.001
**Work Hour**		84	-1.807	0.514	<0.001	-2.276	0.584	<0.001
**Walk Distance Day**		84	-0.001	<0.001	0.002			
**Walk Time Day**		84	-0.034	0.009	<0.001			
**Chain Hour**		84	0.082	0.121	0.500			
**Rest Day**								
	Shed	47	Reference					
	Open area	30	-3.792	1.947	0.052	-5.975	3.537	0.091
	Tree	7	0.042	3.433	0.990	4.353	4.376	0.320
**Rest Night**								
	Shed	38	Reference					
	Open area	21	-1.250	2.380	0.600	1.640	3.170	0.605
	Enclosure	14	-1.330	2.460	0.590	-7.414	6.238	0.235
	Forest	8	-13.660	2.350	<0.001	-14.329	5.375	0.008
	Tree	3	-10.550	4.360	0.016	-8.564	4.348	0.049
**Free Foraging**								
	Yes	33	Reference					
	No	51	2.350	1.940	0.230			
**Ankus**								
	Yes	44	Reference					
	No	40	1.430	1.910	0.450			

SE = Standard error

Variables having a *P* value < 0.15 at the univariate analysis were included in the multivariable analysis.

LS-mean FGM concentrations in relation to categorical demographic and management variables significant in the final GEE models are presented in Table 5. Concentrations of FGM were lower in females than males (*P* = 0.001). Elephants in the observation only program had higher FGM compared to elephants that participated in riding with a saddle programs (*P* = 0.003) and shows (*P* < 0.001). Elephants that were kept in open areas had lower FGM concentrations compared to elephants that were isolated under a tree (*P* = 0.003). Elephants that were kept in the forest at night had lower FGM concentrations compared to elephants that were kept in open areas inside the camps (*P* = 0.017).

**Table 5:** LS-mean (± SEM) fecal glucocorticoid metabolite (FGM) concentrations of elephants in relation to categorical demographic and management variables from final GEE models.

Variable		N	FGM (ng/g)
**Sex**			
** **	Male	18	56.5±2.42^b^
** **	Female	66	47.7±1.87^a^
**Work Type**			
** **	Observation	4	67.4±5.03^c^
** **	Riding with a saddle	22	46.8±2.46^ab^
** **	Riding bareback	37	53.4±2.55^bc^
** **	No riding	19	59.9±3.41^c^
** **	Show	2	33.2±4.86^a^
**Rest Day**			
** **	Shed	47	52.7±2.55^ab^
** **	Open area	30	46.7±2.07^a^
** **	Tree	7	57.0±3.42^b^
**Rest Night**			
** **	Shed	38	57.9±3.52^ab^
** **	Open area	21	59.4±3.43^b^
** **	Enclosure	14	50.5±3.53^ab^
** **	Forest	8	43.6±2.99^a^
** **	Tree	3	49.3±4.84^ab^

^a,b,c^Different letters indicate significant differences within each variable category (*P* < 0.05).

Results of univariate and multivariable GEE analyses of stereotypic behaviors, BCS, FS, and WS associated with FGM are presented in Table 6. All variables significantly affected FGM in the univariate analysis. In the final multivariable analysis, the combination of stereotypic behaviors, FS, and WS significantly affected FGM. From the orthogonal polynomial models, there were positive linear effects of BCS and WS on FGM in the univariate analysis (S4 Table).

**Table 6:** Univariate and multivariable GEE analyses of stereotypic behaviors, BCS, FS, and WS associated with mean (± SEM) fecal glucocorticoid metabolite (FGM) concentrations of elephants in each category.

Variable		N	Univariate analysis			Multivariable analysis		
			Estimate	SE	*P* value	Estimate	SE	*P* value
**Stereotype**								
	Yes	21	Reference					
	No	63	6.900	1.980	0.001	7.812	2.076	<0.001
**Body condition score**								
	3		Reference					
	2		-2.500	5.240	0.633	-4.952	4.722	0.294
	4		2.820	2.440	0.247	2.474	2.442	0.311
	5		5.130	2.770	0.064	3.836	2.862	0.180
**Foot health score**								
	0		Reference					
	1		-1.880	2.251	0.404	-2.151	2.283	0.346
	2		-5.599	2.499	0.025	-7.141	2.610	0.006
	3		0.349	10.237	0.973	0.953	2.590	0.907
**Wound score**								
	0		Reference					
	1		0.778	2.731	0.776	2.041	2.650	0.441
	2		7.359	3.946	0.062	8.428	3.912	0.031

SE = Standard error

Variables having a *P* value < 0.15 at the univariate analysis were included in the multivariable analysis.

LS-mean FGM concentrations in association with stereotypic behavior, BCS, FS, and WS categories that were significant in the multivariable GEE models are presented in Table 7. Elephants that did not show stereotypic behaviors had higher FGM than elephants that did (*P* < 0.001). Elephants with a FS of 2 had lower FGM than elephants with a FS of 0 (*P* = 0.032). There were no differences in LS-mean FGM based on BCS or WS (*P* > 0.05).

**Table 7:** LS-mean (± SEM) fecal glucocorticoid metabolite (FGM) concentrations of elephants in each category of stereotypic behaviors, BCS, FS, and WS from multivariable GEE models.

Variable		N	FGM (ng/g)
**Stereotype**			
	Yes	21	48.1±4.07^a^
	No	63	55.9±3.25^b^
**Body condition score**			
	3		51.6±3.75^a^
	2		46.7±5.63^a^
	4		54.1±3.44^a^
	5		55.5±3.64^a^
**Foot health score**			
	0		54.2±2.42^b^
	1		52.1±2.23^ab^
	2		47.1±2.45^a^
	3		54.5±11.69^ab^
**Wound score**			
	0		48.5±3.33^a^
	1		50.5±3.86^a^
	2		56.9±5.05^a^

^a,b^Different letters across columns indicate significant differences for each variable (*P* < 0.05).

## Discussion

This is the first large scale study to examine the effect of management practices on FGM concentrations in tourist elephants using an epidemiological approach. We identified several relationships between FGM and welfare indicators that shed light on how tourism affects physiological function. An earlier camp survey suggested the majority of captive elephants in Thailand experience poor welfare [47]; however, conclusions were based on subjective criteria and not correlated with any biological welfare indices. In our study, work activities like riding with a saddle or shows were actually associated with lower FGM concentrations, and neither chaining nor use of ankus had significant effects on stress levels of elephants in this population. In a related study of tourist elephants in Thailand, exercise in the form of riding was associated with better BCSs, lower FGM concentrations and healthier lipid and metabolic profiles [31]. Thus, it may be too simplistic to assume that all tourist activities are universally bad for elephants. And in fact, elephants in the one observation only camp had the highest FGM concentrations. Those elephants had limited interactions with mahouts and tourists, but they were in very close proximity with people (within a few meters) all day for observation. Activity was primarily standing, with limited time for walking or socializing.

Burn [48] discusses how wild and domesticated animals in captivity can suffer because of chronic inescapable boredom that in some situations can negatively affect neural, cognitive and behavioral flexibility. Interactions with humans can be beneficial and reduce stress in many species. For example, dogs whose owners regard their animals as social partners or meaningful companions have relatively low salivary cortisol concentrations [49]. In elephants, positive keeper interactions can promote good welfare [50]. In that study, for keepers handling Asian elephants in North American zoos, a high score on the attitude variable ‘keeper as herdmate’ predicted low serum cortisol in elephants. Additionally, keepers that felt accepted as part of the herd, spent more time talking to their elephants, and believed they had special bonds with them worked with elephants that had overall lower cortisol. Rossman et al. [51] investigated how captive African elephants chose to initiate interactions with humans and found several showed preferences in interacting with specific guides, indicating good elephant-keeper bonds. The need for dominance hierarchies is common in social species, including elephants [52]. Although the observation camp promoted free roaming, the creation of bonded social groups is rare. And in fact, elephants at one of the observation camps had the highest FGM concentrations, which could be from a high density of elephants in a limited area. Mahouts there do not carry an ankus, and intra-elephant aggression is not uncommon. Elephants at that camp also get less exercise and are quite obese (median BCS = 5), a factor associated with poor health outcomes [31].

In the univariate analysis, *Walk Distance*, *Time Day*, and *Work Hour* all were associated with lower FGM, with *Work Hour* remaining in the final model. Besides being good exercise, work and walking can also serve as enrichment that prevents boredom in elephants. Studies in rats, mice, and humans have shown the beneficial effects of a physically active life style (regular voluntary exercise) on HPA activity, sleep physiology, and cognitive and anxiety-related behavior. By contrast, forced exercise can result in a significantly higher GC response [53]. Recently, Norkaew et al. [32] found notable camp differences in elephant FGM, BCSs and metabolic health that suggests management practices can affect physiological function and welfare; some negatively like overfeeding of high calorie treats, others positively, such as exercise, even in the form of riding. Thus, work activities can benefit elephant health and welfare, but only if properly controlled.

During the night, elephants that were restrained in the forest under a tree canopy had lower FGM than elephants that were kept in open areas inside the camps. Unnatural environments can be sources of stress if they do not provide animals with opportunities to interact with surroundings in ways that promote the development of sensory and cognitive abilities, or allow display of species-typical behaviors [54]. In zoos, environmental enrichment adds biological complexity and reduces stress by providing animals with more behavioral options [55]. Environmental enrichment also increases walking behavior, reduces foot problems, and promotes normal ovarian function and hormone status [34, 56, 57]. Forests provide elephants with more natural environments and offer opportunities to explore and forage, even when chained. Most (91%) elephant camps kept elephants on chains at night due to lack of suitable enclosures, and only about 3% secured elephants in the forest [2], so we hope our findings will encourage more facilities to manage elephants under more natural conditions, at least at night. There were no wild elephants near the camps in this study; however, in other areas, chained elephants have been attacked by wild elephants, which would be a concern.

During the day, elephants kept in open areas had lower FGM than those restrained under a tree canopy. Although trees provide adequate shade, elephants are usually kept on short chains as a safety measure, which can prevent tactile interactions with conspecifics. By contrast, free roaming in open areas does provide opportunities for elephants to socialize and interact. Vocalizations, chemical signals, and visual and tactile displays all play a role in short‐distance interactions of elephants [58]. More importantly, elephants are highly tactile and interactive, and conspecific affiliation is an important part of social integration [59, 60]. Therefore, interactions without tactile behavior may limit their social relationships, and by extension their welfare. For many animals in captivity, social isolation is clearly stressful [54], and exposure to social isolation stress can induce activation of the hypothalamic–pituitary–adrenal axis and release of GCs [61]. Studies in elephants also found that spending time housed separately [24] or chained [62–64] increased the risk of performing frustration behaviors; i.e., stereotypy.

Increasing age was found to be associated with lower FGM in the univariate analysis, but only trended towards significant (*P* = 0.059) in the multivariable model. Similar to our study, Kumar et al. [8] found the lowest FGM concentrations in one old female and the highest in one of the younger females in captive Asian elephants in south Indian zoos. By contrast, Brown et al. [6] reported an increase in serum cortisol with age in captive bull elephants in the U.S.. Still other studies of zoo [65] and free-ranging [66–68] elephants observed no significant influence of age on FGM concentrations. It is difficult to determine the significance of age effects on the HPA axis as they are confounded by intrinsic and extrinsic factors that likely depend on the environments in which the elephants live. Older animals are often calmer and more tolerant to handling [69], which could lead to less intensive control by keepers/mahouts. In addition, older elephants might cope better with their environment through learning and adaptation to daily schedules. In most camps, older elephants generally have a reduced work load or are kept in separate geriatric camps that provide targeted care and attention to specific needs (e.g., [2]).

Female elephants had lower FGM than males, which is similar to a study of Woolley et al. [70] that found higher FGM in male compared to female free-ranging African elephants. Tingvold et al. [71], however, found the opposite: FGM concentrations were lower in male than female free-ranging African elephants. It has been speculated that elevated stress hormone concentrations in female elephants reflect the added responsibility of caring for juveniles and calves, and also perhaps to added nutritional and biological needs of being pregnant and/or lactating [72]. Glucocorticoid secretion can be influenced by any number of factors, although in this study, environmental conditions were the same for both sexes; none of the females were pregnant, and males were non-musth. However, bulls often require more control through ankus use or chaining because of more aggressive behaviors, which could explain the higher FGM in this population.

One unexpected finding was that elephants that presented with stereotypic behaviors had lower FGM than elephants that did not, which contrasts with other studies that found stereotypies were most often associated with elevated GC concentrations [25, 73]. As pointed out by Pomerantz et al. [74], however, there are similarities between stereotypy in captive animals and humans, including the involvement of neurological malfunctions that lead to the expression of aberrant behaviors. One hypothesis is that stereotypic behavior functions as a general ‘coping mechanism’, increasing or decreasing arousal, to cope with stressful environments. ‘Coping’ covers a broad range of responses and behaviors, both learned (e.g. active avoidance) and unlearned (e.g., hiding; fighting; displacement activities) that can have either very specific effects (e.g., reducing the HPA response to a specific stressor) or act as a ‘general panacea’ (i.e., attenuating negative subjective states in any adverse situation). The coping effects associated with performing a source behavior are hypothesized to reinforce it, thereby leading to the repetitive performance typical of stereotypies [75, 76]. More recently, using neuropsychological tests and measures of FGM concentrations in captive rhesus macaques, stereotypies were found to be related to brain pathology, and an adaptive mechanism to better cope with stress [74]. Some stereotypies in those monkeys were positively correlated with perseveration (e.g., self-directed and fine motor), suggesting basal ganglia malfunction, while self-directed stereotypies also were negatively correlated with an increase in FGM concentrations following a stress challenge, thus representing a coping mechanism. Wiepkema et al. [77] studied the relationship between abomasal damage and stereotypies of crated veal calves and found that those animals that exhibited tongue-playing stereotypies had no ulcers, while calves that did not perform that behavior did. Briefer Freymond et al. [78] showed that crib-biting in horses reduced cortisol concentrations, and further suggested that preventing the behavior would limit the ability of individuals to cope with perceived stressors. The lower FGM concentrations in elephants that stereotyped in our study supports the ‘coping hypothesis’ [75], although there is little argument that these behaviors develop under suboptimal conditions and should be mitigated by improved management.

We expected foot problems to contribute to higher FGM concentrations. In horses and cattle, hoof pain and inflammation (laminitis) are clearly associated with increased cortisol secretion [79, 80]. However, elephants with moderate foot problems (FS = 2) actually exhibited lower FGM than those with no foot problems (FS = 0), which suggests there may be other covariates or confounding factors that influence FGM concentrations apart from foot pain. Similarly, elephants with severe foot problems exhibited FGM that were no different from FS = 0, although a FS of 3 was only observed six times, thus limiting our ability to identify significant effects. We questioned whether these lower concentrations might reflect a hypo adrenal state, but found concentrations were well within the range of other studies utilizing the same immunoassay, if not on the higher end [31, 41].

Use of an ankus had no significant effect on FGM concentrations in the model; however, misuse of the ankus is definitely a welfare concern. Wounds were noted in half of the elephants (42 of 84) caused primarily by use of an ankus (63% of the observations, n = 41), but also by knives, scratches, and pressure sores. In a larger study by Bansiddhi et al. [35], ankus wounds included abrasions, lacerations, ulcers, and abscesses, while knives caused both penetrating and incision wounds. Knives are primarily used to cut grass or food for elephants, but some mahouts use them to control their elephants. Other injuries in that study included those caused by saddles and saddle ropes, and by intra-specific fighting (2.1%), although those observations could not be included in this study because of incomplete sample sets. In both univariate and multivariable models, elephants with major wounds (WS = 2) had higher FGM than elephants with no visible wounds (WS = 0), which was hypothesized. Using an ankus was found to be associated with higher WS in a larger study in Thailand [35]. Other studies [81, 82] also found elevations in FGM concentrations in free-ranging African elephants with physically injuries. In humans, acute and chronic wounds can cause considerable discomfort, which is a stressor. Pain can result from the wound itself, and also treatments for the underlying condition [83, 84].

In the univariate analysis, elephants with a BCS = 5 (obese) had higher FGM than those with a BCS = 3 (ideal/normal), although a similar study with a smaller number of elephants did not find such a relationship [31]. By contrast, a study of free-ranging Asian elephants by Pokharel et al. [67] found lower FGM with higher BCSs, but that was due to concentrations being highest in individuals with the lowest BCS (BCS = 1), with FGM for BCS = 3, 4 and 5 being similar. Thus, being too fat or too thin may be associated with higher stress hormone concentrations in elephants depending on the situation. In the study of Norkaew et al. [31], although not correlated with BCS, FGM concentrations were positively related to a number of health measures, including total cholesterol, high density lipoproteins, glucose, and insulin. In humans, elevated and sustained cortisol secretion during chronic stress can lead to central obesity, hypertension, glucose intolerance, and dyslipidemia [85, 86].

In conclusion, measures of FGMs can inform on the well-being of tourist elephants, although it is best done in combination with other physiological, health, and behavioral measures. Our aim was to base conclusions on evaluations of accepted welfare indicators rather than to rely on subjective assumptions of what is good or bad for elephants. To minimize stress, this study suggests that elephants benefit from participating in walking or work activities (i.e., exercise), resting in natural environments, and socializing with conspecifics. It also was evident that a ‘no hook, no ride’ policy may not always be best for welfare, especially if the alternative activities rely on over feeding of non-nutritious foods, and restricted activity that creates boredom and does not encourage natural social interactions. There was considerable variation across camps in FGM and welfare measures, so more work is needed to determine how specific husbandry and management strategies affect welfare of elephants at the individual level. Ultimately, the goal is to develop science-based guidelines to aid in the management of elephants under human care in Thailand for long-term sustainability of captive elephants throughout southeast Asia.

## Supporting information

S1 Table Scoring system for assessing foot health. (DOCX)

S2 Table Scoring system for assigning wound scores. (DOCX)

S3 Table The bivariate correlation table for the continuous variables in the univariable analysis. (DOCX)

S4 Table Orthogonal polynomial models for univariate and multivariable GEE analyses of BCS, FS, and WS associated with mean (± SEM) fecal glucocorticoid metabolite (FGM) concentrations of elephants. (DOCX)

S5 Table Dataset of demographic, management variables, and results of welfare indicators (i.e., FGM, BCS, FS, WS, and stereotypic behaviors) of elephants. (XLSX)
