# Supplementary material for: Management factors affecting adrenal glucocorticoid activity of tourist camp elephants in Thailand and implications for elephant welfare
Source: PLoS One. 2019 Oct 1;14(10):e0221537. doi: 10.1371/journal.pone.0221537 (PMC6771993; doi:10.1371/journal.pone.0221537)
Supplement: S1 Table — (DOCX) [file pone.0221537.s001.docx]

**S1 Table.** Scoring system for assessing foot health adapted from Harris et al. [37] and the British and Irish Association of Zoos and Aquariums (BIAZA) Elephant Welfare Group as described by Todd [38].

| Score | Description |
| --- | --- |
| 0 (normal) | No lesions, normal nails |
| 1 (mild) | Uncomplicated nail cracks (small cracks which did not extend into the cuticle), mild overgrowth of nails or cuticles, mild dry cuticles, mild disfigured nails or mild injuries |
| 2 (moderate) | Complicated nail cracks (nail cracks exposing underlying tissue), moderate overgrowth of nails or cuticles, moderate dry cuticles, infection or moderate injuries |
| 3 (severe) | Underlying tissues exposed plus evidence of purulent discharge, deep pododermatitis, nail loss or severe injuries |
