# Supplementary material for: Management factors affecting adrenal glucocorticoid activity of tourist camp elephants in Thailand and implications for elephant welfare
Source: PLoS One. 2019 Oct 1;14(10):e0221537. doi: 10.1371/journal.pone.0221537 (PMC6771993; doi:10.1371/journal.pone.0221537)
Supplement: S2 Table — (DOCX) [file pone.0221537.s002.docx]

**S2 Table.** Scoring system for assigning wound scores based on Schein et al. [39].

| Score | Description |
| --- | --- |
| 0 | No lesions |
| 1 (minor) | Minor wounds such as scrapes, scratches, superficial wounds or mild bleeding, some serous discharge |
| 2 (major) | Major wounds such as severe bleeding, severe infection with pus, deep destruction of tissue, exposing muscle or bone |
