# Supplementary material for: Management factors affecting adrenal glucocorticoid activity of tourist camp elephants in Thailand and implications for elephant welfare
Source: PLoS One. 2019 Oct 1;14(10):e0221537. doi: 10.1371/journal.pone.0221537 (PMC6771993; doi:10.1371/journal.pone.0221537)
Supplement: S3 Table — (DOCX) [file pone.0221537.s003.docx]

**S3 Table.** The bivariate correlation table for the continuous variables in the univariate analysis.

| Variable | Age | Work Hour | Walk Distance Day | Walk Time Day | Chain Hour |
| --- | --- | --- | --- | --- | --- |
| Age | 1 |  |  |  |  |
| Work Hour | -0.22* | 1 |  |  |  |
| Walk Distance Day | -0.25* | 0.61* | 1 |  |  |
| Walk Time Day | 0.12 | 0.44* | 0.30* | 1 |  |
| Chain Hour | -0.26* | 0.06 | 0.20 | -0.22* | 1 |

*Significant at 0.05.
