# Supplementary material for: Management factors affecting adrenal glucocorticoid activity of tourist camp elephants in Thailand and implications for elephant welfare
Source: PLoS One. 2019 Oct 1;14(10):e0221537. doi: 10.1371/journal.pone.0221537 (PMC6771993; doi:10.1371/journal.pone.0221537)
Supplement: S4 Table — (DOCX) [file pone.0221537.s004.docx]

**S4 Table.** Orthogonal polynomial models for univariate and multivariable GEE analyses of BCS, FS, and WS associated with mean (± SEM) fecal glucocorticoid metabolite (FGM) concentrations of elephants.

| Variable |  | N | Univariate analysis | | |  | Multivariable analysis | | |
| --- | --- | --- | --- | --- | --- | --- | --- | --- | --- |
|  |  |  | **Estimate** | **SE** | ***P* value** |  | **Estimate** | **SE** | ***P* value** |
| Body condition score | | |  |  |  |  |  |  |  |
|  | Linear | | 5.750 | 3.538 | 0.100 |  | 6.072 | 3.312 | 0.067 |
|  | Quadratic | | -0.093 | 2.876 | 0.970 |  | 0.006 | 2.746 | 0.998 |
|  | Cubic | | -0.183 | 2.007 | 0.930 |  | -0.210 | 1.973 | 0.915 |
| Foot health score | | |  |  |  |  |  |  |  |
|  | Linear | | -0.598 | 6.886 | 0.930 |  |  |  |  |
|  | Quadratic | | 3.914 | 5.245 | 0.460 |  |  |  |  |
|  | Cubic | | 2.573 | 2.757 | 0.350 |  |  |  |  |
| Wound score | | |  |  |  |  |  |  |  |
|  | Linear | | 5.200 | 2.790 | 0.062 |  | 5.576 | 2.901 | 0.055 |
|  | Quadratic | | 2.370 | 2.600 | 0.363 |  | 1.926 | 2.650 | 0.467 |

SE = Standard error

Variables having a *P* value < 0.15 at the univariate analysis were included in the multivariable analysis.
